# Routine failures in the process for blood testing and the communication of results to patients in primary care in the UK: a qualitative exploration of patient and provider perspectives

**DOI:** 10.1136/bmjqs-2014-003690

**Published:** 2015-08-06

**Authors:** Ian Litchfield, Louise Bentham, Ann Hill, Richard J McManus, Richard Lilford, Sheila Greenfield

**Affiliations:** 1 School of Health and Population Sciences, Medical and Dental Sciences, University of Birmingham, Birmingham, UK; 2 Department of Transformation, Worcestershire Acute Hospitals, Worcestershire, UK; 3 Nuffield Department Primary Care Health Sciences, University of Oxford, Oxford, UK; 4 Health Sciences, Warwick Medical School, University of Warwick, Coventry, UK

**Keywords:** General practice, Healthcare quality improvement, Patient-centred care

## Abstract

**Background:**

The testing and result communication process in primary care is complex. Its successful completion relies on the coordinated efforts of a range of staff in primary care and external settings working together with patients. Despite the importance of diagnostic testing in provision of care, this complexity renders the process vulnerable in the face of increasing demand, stretched resources and a lack of supporting guidance.

**Methods:**

We conducted a series of focus groups with patients and staff across four primary care practices using process-improvement strategies to identify and understand areas where either unnecessary delay is introduced, or the process may fail entirely. We then worked with both patients and staff to arrive at practical strategies to improve the current system.

**Results:**

A total of six areas across the process were identified where improvements could be introduced. These were: (1) delay in phlebotomy, (2) lack of a fail-safe to ensure blood tests are returned to practices and patients, (3) difficulties in accessing results by telephone, (4) role of non-clinical staff in communicating results, (5) routine communication of normal results and (6) lack of a protocol for result communication.

**Conclusions:**

A number of potential failures in testing and communicating results to patients were identified, and some specific ideas for improving existing systems emerged. These included same-day phlebotomy sessions, use of modern technology methods to proactively communicate routine results and targeted training for receptionists handling sensitive data. There remains an urgent need for further work to test these and other potential solutions.

## Introduction

The reasons for ordering blood tests in primary care are varied, and yet, the rapid and accurate communication of results remains central to ensuring patients receive timely and appropriate care.^1^ As the numbers of tests ordered in primary care continue to increase, there is a need for increasingly flexible, yet robust, systems for managing testing and result communication. The total testing process (TTP) is complex, encompassing test ordering, phlebotomy, dispatch of sample, dissemination of results and the initiation of appropriate follow-up.^2^ The success of the TTP is potentially hindered by the absence of satisfactory guidelines, and relies on a range of practice staff (including those without clinical expertise), external groups in laboratory and hospital settings, and patients. Errors in the process can lead to serious harm for patients,^1^ ^3–6^ and medicolegal concerns for healthcare providers.^7–9^ General practices in the UK recently identified the handling of test results as one of the top 10 risks for patient safety,^10^ with some reporting that up to one-third of patients are not notified of abnormal results.^4^

In analysing the TTP in the USA, errors have been attributed to a number of social and organisational factors at practice level, these include documentation errors, delays in responding to clinical information, difficulties in contacting patients, time constraints of practice staff and forgetfulness.^4^ There is evidence that these errors are compounded by the limited training of practice staff and a lack of awareness of the scale of the problem.^11^ Recent studies report that only half of family practices have written protocols for result management.^5^ In particular, there was no agreed set of operating procedures describing what was to be done if the result was positive, or for allowing the patient some discretion over the method by which results would be communicated.^6^ In response, there are increasing calls for a more unambiguous process, linking the ordering of a test, the mechanism by which the results may be communicated and, if appropriate, further action being initiated.^3^ ^6^ ^12–14^ It is apparent that any system redesign must reference the perspectives and preferences of all involved in the process, and that using methodology such as ‘experience-based co-design’ means that patients’ preferences can be reconciled with available resources.^13^ ^14^

To date, only one other study based in the UK has explored the TTP from either staff or patient perspectives.^15^ Due to this, little is known of what works well in NHS general practices or which aspects of the process can be usefully improved. Here, we collate data from a series of focus group discussions with patients and staff in order to assess strengths and weaknesses of current systems, and identify areas and develop strategies for improvement that account for patient preference, staff capabilities and logistical feasibility.

## Methods

Four general practices were selected from 10 previously collaborating in Birmingham and Lambeth Liver Evaluation Testing Strategies (BALLETS), a prospective study of abnormal liver function tests in England.^16^ During BALLETS, we discovered that methods of test result communication varied between practices. Judgement sampling, based on our knowledge of the practices, was used to purposively select four practices to take part in focus groups. These practices reflected a range of size, socioeconomic environment and communication pathways encompassing a range of overlapping methods and systems (see table 1).^17^

**Table 1:** Breakdown of general practices where participating patients are registered

General practice study ID	Number of patients registered	Number of full-time equivalent GPs	IMD code*
Practice 1	23 727	7.3	15 066
Practice 2	5914	3.0	13 866
Practice 3	7059	6.3	871
Practice 4	27 430	12.3	8447

*Index of multiple deprivation (IMD) ranking out of 32 482 lower super output levels in England. The IMD codes, produced by the UK Government and first released in 2004 and updated in 2010, provide indicators of deprivation in local authority areas to inform health and social policy.^18^

**Figure 1:**
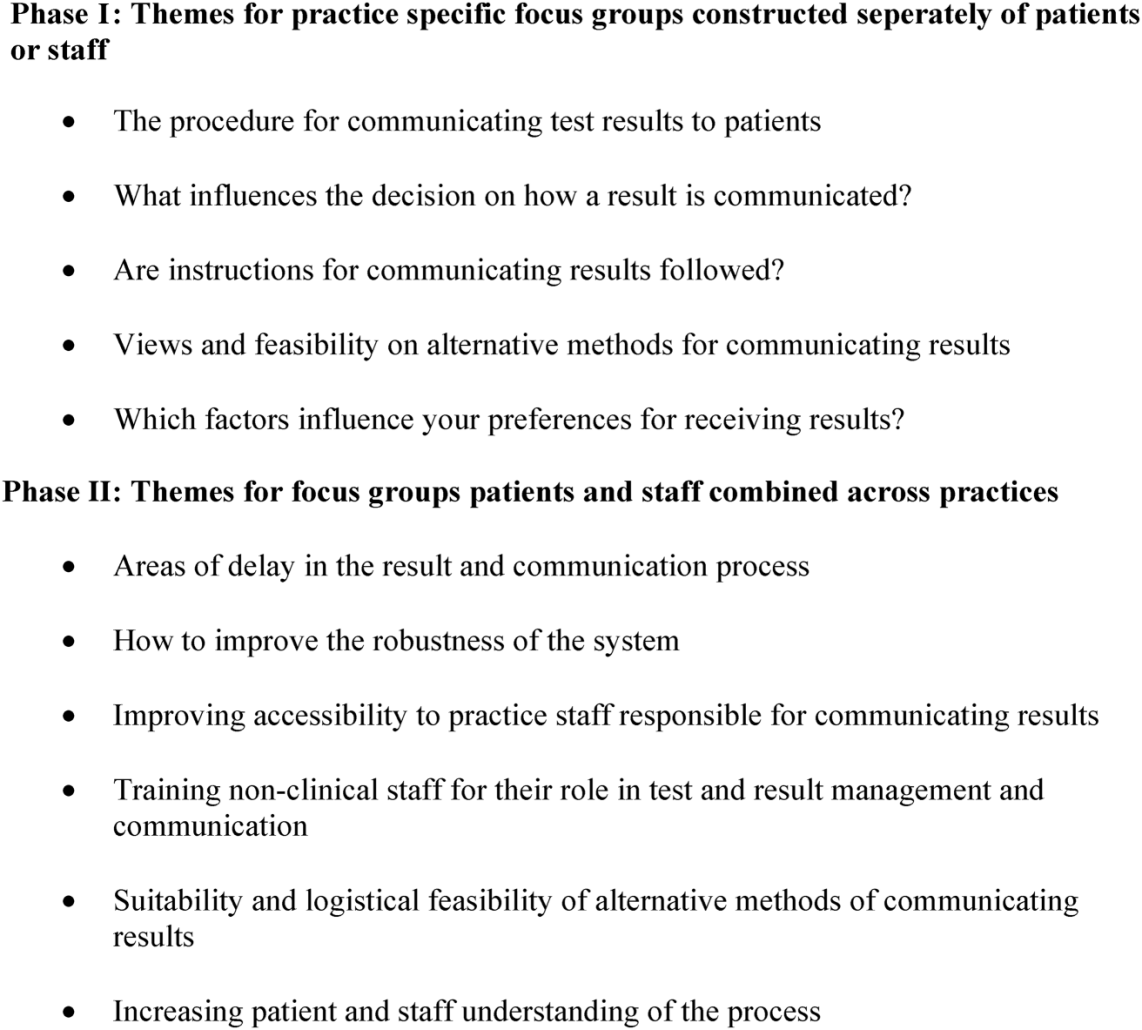
Themes for discussion for Phase I and Phase II focus groups.

Focus groups were conducted in two phases. The first phase of groups was practice specific, and we met with staff and patients separately (see online supplementary appendix table S1).^19^ Participants in staff focus groups were selected from all staff currently involved in the communication of test results; general practitioners, practice nurses, healthcare assistants, receptionists and practice managers. Participants in patient focus groups were drawn from those with experience of receiving test results, while the groups maintained the maximum variability of patient characteristics, such as age, gender and ethnicity to include a range of opinions and experiences. The topic guides explored what staff and patients perceived to be the strengths and weaknesses of their existing system, the role of patients and staff, how both groups felt the service could be improved, alternative methods for communicating test results in primary care, including patient preferences and how they might best be accommodated (see figure 1 for themes explored).

Each focus group was attended by a moderator and a researcher who acted as an observer and took down field notes to be used in conjunction with the digitally recorded discussions, which were then transcribed verbatim. Each transcript was examined closely, and the findings analysed thematically by IL, LB and SG who met and agreed on emerging themes to decide on a coding framework. Transcripts were analysed alongside the field notes, using constant comparative analysis.^20^

The group discussions from Phase I were used to create a process map first. These maps display a set of activities (and their respective values) as a series of steps that are involved in creating a product or providing a service, and are fundamental to process-improvement methodologies such as ‘lean’. Each map provides the opportunity to understand interaction between steps, the roles of various individuals and the flow of materials and information required to provide a service.^21–23^ The detailed process map allowed us to create a service blueprint, a tool originally used in the service industry to diagnose problems with operational inefficiency and now increasingly used in the healthcare environment to drive service innovation.^24^ ^25^ Our blueprint was to focus on the process from the different perspectives of general practitioner (GP) and patient, identifying areas of delay and failure in the process.

The second phase consisted of focus groups, comprised of both staff and patients combined from all four participating practices, some of whom had participated in Phase I group discussions (see online supplementary appendix table S2). The topic guide for these discussions was informed by the findings of Phase I, including the service blueprint, and included suggestions for improving the current system (see figure 1). Groups were again attended by a moderator and a researcher who acted as observers, and the same individuals and method of analysis as used in Phase I were employed.

## Results

From our discussions with staff and patients, it became apparent that those urgent tests with potentially serious implications were followed closely by the GP and patient. However, for the vast majority of diagnostic blood tests, the following default pathway for communicating results emerged. Following the decision to order a test, the patient meets with a phlebotomist to provide a blood sample, which is then collected for testing by a laboratory located within a large local hospital. Following testing, the result is issued to practices electronically using the Pathology Messaging Implementation Programme (PMIP) standard.^26^ Typically, patients are asked to telephone practices 7 days after providing a sample to learn their result and to arrange any appropriate follow-up.

### Service blueprint

**Figure 2:**
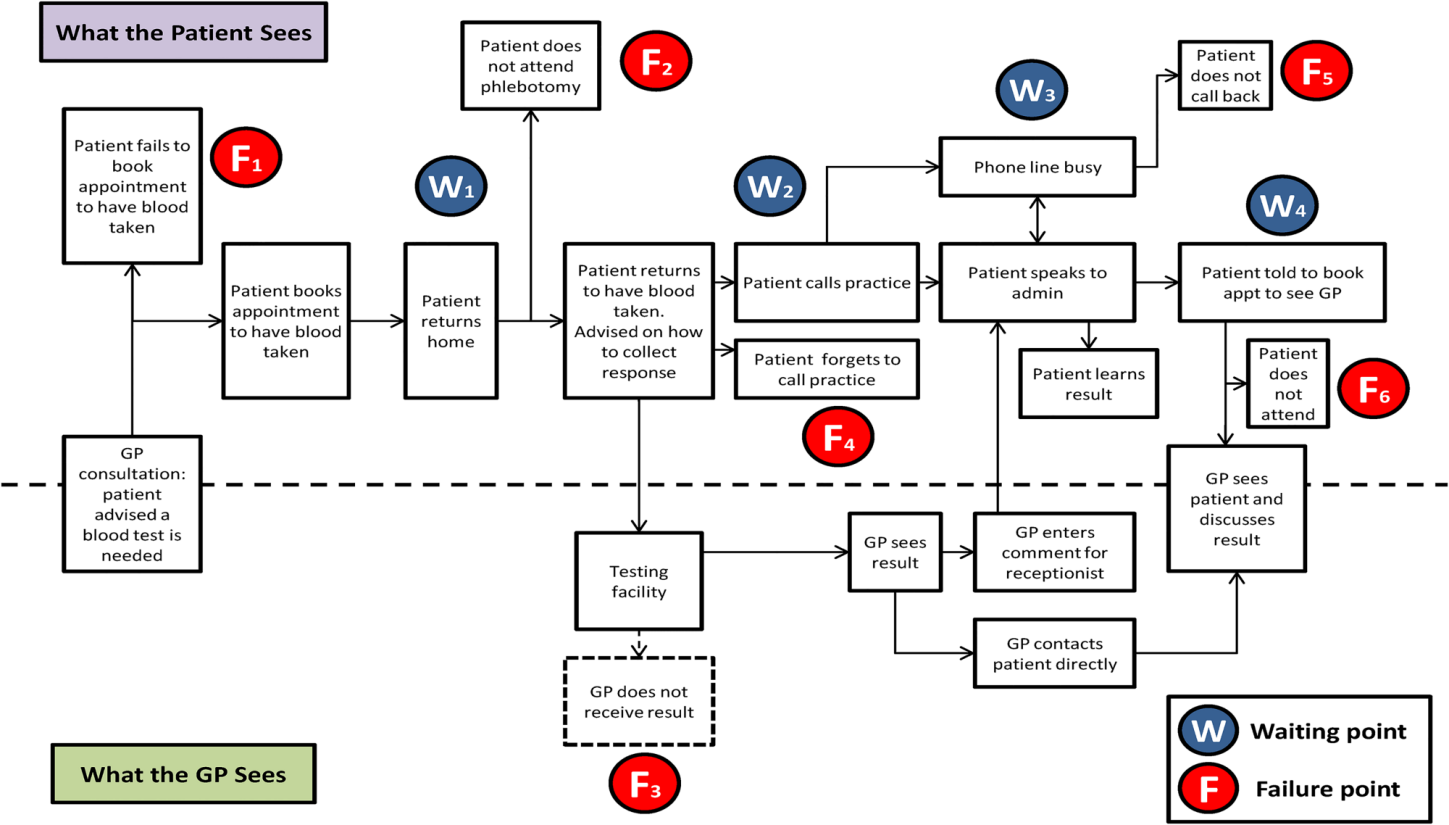
Service blueprint for (diagnostic) blood test communication in primary care.

From patient and staff accounts, we were able to create a service blueprint of the components of the TTP using the example of a diagnostic blood test (figure 2). The blueprint shows the twin perspectives of GP and patient, and locates both areas of delay (waiting points) and where the process can fail (failure points).

The GP has fewer steps to take in the process than patients who may be required to make repeat visits to the practice, and deal with a variety of staff when they do so. Four key sources of delay were identified, and are primarily visible to patients. The first the wait for phlebotomy (W1) and second the time taken to analyse the sample (W2). The third and fourth waiting points concern retrieving results, either via busy phone lines (W3) or in person via the GP (W4).

There were six areas identified where the process may fail, only one of which was visible to GPs—the failure to receive the result from the laboratory. The other five locations of potential failure were associated with patients failing to take appropriate action at the correct time. This ranged from booking an appointment with the phlebotomist (F1) to failing to attend an appointment with the GP to receive their result (F6). A total of six areas within the TTP emerged where improvements could be usefully and practically implemented. These consisted of reducing delay prior to blood sampling, a fail-safe to detect missing and delayed results, improvement in managing calls from patients seeking results, addressing the role of non-clinical staff in result communication, routine communication of non-critical results by the practice and defining and disseminating among patients and staff a protocol for the TTP. Taken together, these improvements will facilitate a more efficient and expeditious route for patients to access results.

### Analysis of focus groups

#### Delay in blood sample being taken

Following a GP's decision to order a blood test, they may take a blood sample during the consultation. More typically however, patients are required to visit reception and book an appointment with a phlebotomist or nurse. This usually requires a return visit to the surgery up to 14 days later dependent upon the availability of appointments and the patients’ own schedule.

Well if the doctor says “make an appointment for a blood test” I make it at the reception desk you see, while I'm there, but it's usually a couple of weeks before I can have it. (Phase I Focus Groups: Patient 9, Practice 3)

Patients also expressed concern that making another visit to the practice could have a detrimental impact on their responsibilities elsewhere.

Well the other thing is the cost to the patient. I mean, I'm retired, I can find time for appointments, but if I'm doing a job and you know, tomorrow I've got to go to Germany, and the day after I've got to be somewhere else, taking time out is a cost to the project that I'm working on. (Phase I: Patient 1, Practice 4)

The delay in the blood sample being taken can also provide anxious patients with the opportunity to avoid seeing the phlebotomist.

Patients are scared so they don't turn up [to appointments] and they'll do it continually until we finally do get either hold of them and do it or…that's it, they don't book another. (Phase II: Phlebotomist, Practice 3)

#### Fail-safe

In the current system, laboratories return blood test results to practices electronically using the PMIP format. The specifics of how these results are dealt with varies from practice to practice, some are assigned to the ordering GP while other practices have a central ‘in-box’ where results are collated before being processed by the duty GP. What was common across all practices was that none had a means of detecting when individual results had been returned to the practice from the laboratory, and if so, whether said result had reached the patient. Practices acknowledged that frequently they would be aware of missing results only if prompted by a patient enquiry to look for the result.

You've got no way of knowing that one of those [results] hasn't come back unless the patient rings in to say: “is my blood result back?” (Phase II: Practice Secretary, Practice 2)

It's hard, if the patient hasn't called for the result we may never know that they didn't get the result, especially with an abnormal result. (Phase I: GP, Practice 3)

#### Improve the management of patients telephoning the practice for results

The typical instruction for patients seeking results was to call the practice reception. Practices spoke about the large volume of calls this generates and the time and resources it takes to respond to these calls. Patients also expressed frustration at time spent waiting for their call to be answered on busy lines.

I must say [a queuing system] would help ‘cause I found it just a little annoying that I didn't know where I was in the queue waiting for a response on the phone. (Phase I: Patient 1, Practice 4)

#### Role of non-clinical staff in communicating results

Receptionists were frequently responsible for taking calls from patients seeking results, and used a script written by the GP to provide further information. Patients expressed concerns over the suitability of receptionists to communicate results, particularly those which may have serious consequences.

I would never accept results off the receptionist unless its cholesterol. (Phase I: Patient 23, Practice 1)

Do they have any guidelines about what results they should give? Because there must be some that are not very good for them to [give]. (Phase I: Patient 15, Practice 1)

Patients also felt that receptionists should have an awareness of the potential impact a result may have on patients.

It's not so much about being given the results over the phone as the person giving the results understanding the impact of what they are saying to the person on the end of the phone. (Phase I: Patient 12, Practice 2)

Receptionists are also unable to answer further questions about results or provide clinical information that is not included in the doctor's script. This may remove any reassurance that patients might usually gain from normal results.

In those cases you actually need a straightforward bland, “There is no problem what so ever” then maybe that's OK to get that from the receptionist, but that might not be particularly helpful to you if you still have the ache, the pain, the twinge, and you're not feeling so well … “Did they do the right test?” (Phase I: Patient 13, Practice 2)

The content of the script provided for receptionists by GPs to accompany the result varies depending on the GP. Inconsistencies in the amount of information provided from one test to the next can create anxiety and confusion in patients.

There are some clinicians that like to add lots of comments…some that don't add any comments…one time the result comes back to a doctor who put some very helpful comments…and the next time the result comes back to one of the clinicians who simply used the default comments and doesn't put anything else there. (Phase I: Practice Manager, Practice 4)

#### Routine communication of normal results

Current systems required patients to call the practice for the majority of results. Due to the large number of tests ordered in general practice, relaying normal results in this fashion places a considerable burden on practice resources. Senior staff felt that time spent communicating normal results was a waste of practice resources.

The majority of these [results] are normal, they're fine. They're not results that the doctor has felt the patient needs; that's taking valuable clinical time out. Or even if the senior receptionists are doing it, taking valuable time [rather than getting on with other, perhaps more pertinent tasks] talking about results which are normal. (Phase I: Practice Manager, Practice 4)

Despite practice staff questioning the value of relaying normal results, patients repeatedly expressed a preference for receiving all results.

If you had a test, and there's no fault found as it were, it would be nice to have that confirmed. (Phase I: Patient 1, Practice 4)

We discussed with staff and patients the feasibility of and preferences for alternative methods for routinely communicating normal results that would impact less on practice time and resource yet still meet patient preferences. Patients in our group appeared more comfortable with SMS than staff who felt it was inappropriate or foresaw problems in maintaining accurate records of mobile telephone numbers.

I think text messaging is a good idea for a routine test. (Phase I: Patient 20, Practice 2)

There's an SMS text thing on the pick-up menu and I presume whatever you write in gets texted out. I haven't used it because I don't think it's particularly appropriate. (Phase I: GP 3, Practice 1)

It relies on the mobile numbers being up to date…or the partner's mobile being up to date and things like that, so I'm not a fan of it. (Phase I: GP 2, Practice 1)

#### Protocol of testing and result communication

Staff confirmed that no formal protocols were currently in place for communicating results, and though a default pathway for delivering results existed, it was not consistently adhered to.

Well, here there is a set procedure and the degree to which the clinicians use the procedure is, I think, variable to a degree. The first thing to say is that it is at the clinician's discretion. (Phase I: Practice Manager; Practice 4)

Patients were aware of the general instruction to phone the practice for results, but appeared unaware of what happens in the eventuality of an abnormal test.

… a bit of communication about a system a bit of reassurance—explanation about what will happen if it shouldn't be normal just so you know what to expect. (Phase I: Patient 17, Practice 1)

One of the problems identified by the authors, although not explicitly by focus group members, was that practices only ever gave out instructions for the default method for retrieving results. Therefore, patients were often confused when contacted seemingly ‘out of the blue’ by the practice and informed of their results.

They usually say “we will have your results in a couple of weeks” but I have also been called by the surgery to give me results over the phone, randomly and I thought “why are they calling me?” (Phase II: Patient 12, Practice 2)

## Discussion

### Summary

The accurate, consistent and timely reporting of test results to GPs and then to the patient reduces the likelihood of medical error, and promotes timely and appropriate follow-up. Many services develop over a long period of time in an ad hoc fashion. Such processes are accepted without question, and may even assume a kind of validation through use. Unlike evolution, however, there is no invisible narrative process to ensure that they undergo continuous adaptation and improvement. This is where process and operation management techniques have a role to play as they provide an opportunity to redesign processes that have become increasingly unfit for purpose.^27–29^ In applying aspects of lean methodologies to the process of testing and result management, we located key areas where delay or failure may occur.^21^ ^24^

### Strengths and limitations

Patient participants were drawn from those with recent experience of the testing and result communication process. Where possible, we created groups, which were mixed by age, gender and ethnicity, though younger patients, more likely to be working full time with potentially different needs and expectations, failed to attend focus groups, and as the time available for recruitment was finite, this led to a preponderance of participants aged over 60 years. This is, however, reflective of primary care, where the majority of patients are older adults.

While we cannot claim that the perspectives of patients at the study practices are internationally representative, previous studies in the USA have also found that the process for communicating test results is haphazard and that dissatisfaction with current practice is pervasive.^30^ ^31^

Staff focus groups contained a range of primary healthcare professionals, and there was a range of staff types, gender and ethnicity. That these focus groups consisted of mixed staff grades may have inhibited the openness of some participants. However, creating groups of mixed staff reflected the reality of the practice environment where a range of practice staff interact with each other throughout the process.^32^

We did not explore in depth the use of alternative technologies, for example, the direct communication of results from laboratories to patients or the potential impact on result communication of an increase in point of care testing as these are not yet freely available in the NHS.

Despite the number of focus groups being relatively limited, this is within the range reported in existing literature,^33^ and similar experiences were repeatedly described by both patients and staff across our groups suggesting that we were approaching theoretical saturation.^34^

### Main findings

Both patients and staff acknowledged the unnecessary delay that can be encountered across current communication processes. Timeliness is frequently acknowledged as a key area for improvement,^35^ and reducing delay in healthcare delivery is more typically a matter of improving administration rather than medical science, yet it has the potential to have a large and positive impact on the quality of healthcare. As delay reduction necessitates improving administrative capabilities, lessons may be learned from the experience of time-based management accrued in other industries. For example, by reducing unproductive time (delay), companies in the production and manufacturing industries have been able to reduce costs, improve quality and provide improved services to customers.^36^ In this study, one area where patients repeatedly expressed frustration was the delay experienced between the decision to test and the phlebotomy appointment. Patients reported feeling anxious at the anxiety felt by the subsequent delay in diagnosis and the impact on their time and resources of an additional visit to the practice. In the UK, the importance of an accessible phlebotomy service was previously identified by the Carter Report, which called for samples to be taken at times and in places convenient to patients.^37^ Pilot studies in secondary care have used lean methodologies to improve quality, productivity and patient experience within phlebotomy with notable success.^38^ The same could be true in primary care settings. By reconfiguring provision of appointments to better meet the demand of patient access to phlebotomy can be improved while minimising impact on practice resources.

Missing results have serious implications for patient safety; particularly where patients who hold to the adage that ‘no news is good news’ are unaware of their responsibility for collecting results, or wrongfully assume that practices initiate contact in all instances of abnormal results.^6^ ^30^ ^39^ Despite this, none of the practices we spoke to had a fail-safe in place to ensure that results are returned to staff or patients. The three clinical management systems (Egton Medical Information Systems (EMIS), SystmOne and Invision) currently employed by practices in the UK and two of which by our practices, all possess the capability to collate information on tests ordered, and record the return of results from laboratories. However, staff in our study appeared unclear on the advantages that could be gained, and appeared reluctant to use these features. This hesitancy to engage with health information technology has been observed previously, and has been attributed to a combination of technical factors, which concern the design, usability and functionality of the system,^40^ ^41^ and social factors centred around characteristics of staff, the organisation and the broader socioeconomic environment.^42–47^ Evidence suggests that developers could help overcome some of these barriers, and increase the confidence of customers by sharing a broader knowledge base of the implications of using these systems.^42^ ^47^ If practices are to make the most of the functionality offered by modern software systems then the software needs to possess a demonstrable advantage and be compatible with varying existing methods of work and used by staff comfortable with the technology.^41^ This being the case, practices may be more willing to commit the resources necessary for the relevant staff to be provided with appropriate training. The timely response to phone calls is considered a key feature of patient-centred primary care,^48^ yet the number of tests ordered and the common instruction to call practices for results mean that large volumes of calls are generated, and both staff and patients in our study recognised the difficulty of retrieving results from practice staff via telephone. Previous studies in the UK reported that one-third of practices has too few people answering the phone at peak hours and that ensuing GP ‘phone line jams’ can lead to increased hospital admissions.^49^ ^50^ Greater detail in the time and date given to patients to phone may prevent time wasted on calls for tests not yet completed. In addition, improving the management of practice telephone lines by implementing straightforward measures such as call waiting may help alleviate pressure on practice telephone systems, and reduce the frustration and anxiety of patients waiting on busy lines. There are minimal requirements for the training of practice receptionists,^51^ yet their role is central to a successful testing and result communication process. It became apparent that patients were apprehensive at such a pivotal role being performed by apparently underqualified staff, and during the course of our discussions, three areas of concern emerged.

First, result information relayed by receptionists varied depending on the GP who had written the related script, and often lacked objective data. Receptionists could not provide either supporting information themselves or suggest to patients where supporting information may be located. The surrounding uncertainty about the meaning or accuracy of ‘normal’ results can lead to additional costly and unnecessary medical visits and diagnostic procedures.^52–55^

Second, there is currently no training requirement for receptionists handling confidential or sensitive patient data, and patients questioned the advisability of receptionists relaying results over the phone in busy public areas of the practice.^51^

Third, patients felt reception staff lacked sensitivity when communicating results, and this perception can be related to the style of discourse employed by receptionists. Previous research exploring this issue has identified that receptionists frequently use a task-centred discourse style, which patients can find overly direct, and impact on both a patient's satisfaction and even appointment attendance.^56^ ^57^ Conversely, where reception staff are perceived as friendly (perhaps using a conventionally polite or rapport-building discourse style), patients report greater satisfaction with their staff encounter and the practice as a whole.^58–60^ Previously, the use of well-developed scripts in telephone conversations have been found to increase consistency, save time and facilitate courtesy,^61^ ^62^ and it may be that this dialogue between patients and staff can be improved by providing receptionists with a more structured framework to guide their response.

We found, as have others, that patients preferred to be informed of normal results, and where this preference is met, it can help reduce patient anxiety.^63–66^ Though we acknowledge that electronic methods of communication are not suitable for all patients, our discussions identified SMS as an acceptable method to patients and the most timely and cost-effective way for practices to communicate normal results in advance of the introduction of patients access to online medical records. Despite the potential to reduce the workload of practice staff and improve timeliness in communicating results,^67^ none of our practices yet use SMS to deliver normal results. However, as the model of healthcare delivery in the UK continues to change in line with initiatives designed to place patients at the centre of their care, the use of modern information and communication technologies to communicate normal results appears inevitable.^63^ Both patients and staff recognised that the TTP needed clarification. Many of the downstream problems with test result communication that were discussed can be ameliorated upstream by agreeing beforehand with the patient how the result will be transmitted, and how this may depend on portent of result. For example, if the patient wishes to receive the result by SMS, the mobile phone number can be confirmed at the point of ordering; subsequent actions, contingent on the result, can also be agreed at this point, and patients can be provided information on the implications of the result, providing reassurance and reducing requests for explanation from unqualified staff downstream.^53^ There are a number of ways in which awareness of the TTP can be increased. One such solution discussed in our groups was the provision of patients with a hard copy of salient information during the initial consultation, facilitated by existing clinical systems and informing patients of the tests being undertaken, preventing confusion if the return of results from multiple tests are staggered and clarifying where responsibility lies in the agreed method of communicating results.

## Conclusion

The complex TTP offers practices a number of opportunities for reducing delay and improving efficiency while increasing patient safety and satisfaction. Undoubtedly, the increase in affordability and accuracy of point of care testing means that when it becomes more widespread, it will have a positive impact on many of the issues we highlight here, as will online access to personal health records for patients.^68^ In considering existing systems, delay is a significant cause of anxiety in patients, and can be dramatically reduced by better management of phlebotomy services and practice telephone systems. Introducing a fail-safe to ensure that results have been returned to practices and ultimately to patients will further reduce the risk of delayed or even missed diagnoses. The use of automated systems for returning routine results can release resources to be more productively spent elsewhere. Finally, by providing patients with confirmation of the tests ordered, information on the implications of those tests and the precise route for retrieving results, we can improve engagement, and help ensure the patients remain as complicit as possible in their care. Further work is planned to implement and evaluate practical interventions to improve the TTP in some of these key areas.

## Supplementary Material

Web supplement
