# Supplementary material for: Routine failures in the process for blood testing and the communication of results to patients in primary care in the UK: a qualitative exploration of patient and provider perspectives
Source: BMJ Qual Saf. 2015 Aug 6;24(11):681–90. doi: 10.1136/bmjqs-2014-003690 (PMC4680130; doi:10.1136/bmjqs-2014-003690)
Supplement: Web supplement [file bmjqs-2014-003690-s1.pdf]

Appendix 1

Table 2 Phase One focus group recruitment

**Title**	**Practice One**	**Practice Two**	**Practice Three**	**Practice Four**	**Total**
Staff Focus Group					
General Practitioner	5	7	2	2	16
Practice Manager	1	1	1	1	4
Practice Nurse	2	1	2	1	6
Practice Secretary	0	1	0	0	1
Lead Receptionist	0	1	0	0	1
Healthcare Assistant	1	0	1	1	3
IT lead	1	0	0	0	1
Office manager	1	0	1	1	3
Patient Focus Groups					
Patient	14	9	3	2	28

Table 3 Phase Two focus group recruitment

**FOCUS GROUP ONE**	**FOCUS GROUP TWO**
**Title** Practice **(Gender)**	**Title** Practice **(Gender)**
**Practice Manager** Practice 1 **(Female)**	**Patient 2** Practice 1 **(Female)** *
**Reception Manager** Practice 1 **(Female)**	**Patient 3** Practice 1 **(Male)** *
**Information Technology Lead** Practice 1 **(Male)**	**Office Manager** Practice 3 **(Female)**
**Patient** Practice 1 **(Female)**	**Research Nurse** Practice 3 **(Female)**
**Phlebotomist** Practice 3 **(Female)**	**Patient** Practice 4 **(Male)**
**Practice Secretary** Practice 2 **(Female)**	
**Lead Receptionist** Practice 2 **(Female)**	
**Patient** Practice 4 **(Male)**	
**Patient** Practice 2 **(Male)**	
**Patient** Practice 3 **(Female)**	

**Also took part in Phase One focus groups*
